# Role of Mutation in *Pseudomonas aeruginosa* Biofilm Development

**DOI:** 10.1371/journal.pone.0006289

**Published:** 2009-07-16

**Authors:** Tim C. R. Conibear, Samuel L. Collins, Jeremy S. Webb

**Affiliations:** School of Biological Sciences, University of Southampton, Southampton, United Kingdom; Oxford University, United Kingdom

## Abstract

The survival of bacteria in nature is greatly enhanced by their ability to grow within surface-associated communities called biofilms. Commonly, biofilms generate proliferations of bacterial cells, called microcolonies, which are highly recalcitrant, 3-dimensional foci of bacterial growth. Microcolony growth is initiated by only a subpopulation of bacteria within biofilms, but processes responsible for this differentiation remain poorly understood. Under conditions of crowding and intense competition between bacteria within biofilms, microevolutionary processes such as mutation selection may be important for growth; however their influence on microcolony-based biofilm growth and architecture have not previously been explored. To study mutation *in-situ* within biofilms, we transformed *Pseudomonas aeruginosa* cells with a green fluorescent protein gene containing a +1 frameshift mutation. Transformed *P. aeruginosa* cells were non-fluorescent until a mutation causing reversion to the wildtype sequence occurs. Fluorescence-inducing mutations were observed in microcolony structures, but not in other biofilm cells, or in planktonic cultures of *P. aeruginosa* cells. Thus microcolonies may represent important foci for mutation and evolution within biofilms. We calculated that microcolony-specific increases in mutation frequency were at least 100-fold compared with planktonically grown cultures. We also observed that mutator phenotypes can enhance microcolony-based growth of *P. aeruginosa* cells. For *P. aeruginosa* strains defective in DNA fidelity and error repair, we found that microcolony initiation and growth was enhanced with increased mutation frequency of the organism. We suggest that microcolony-based growth can involve mutation and subsequent selection of mutants better adapted to grow on surfaces within crowded-cell environments. This model for biofilm growth is analogous to mutation selection that occurs during neoplastic progression and tumor development, and may help to explain why structural and genetic heterogeneity are characteristic features of bacterial biofilm populations.

## Introduction

Autonomously replicating cells under conditions of cellular crowding, for example within eukaryotic malignancies, are constrained by intensely competitive and nutrient limited growth conditions. In these circumstances microevolutionary processes, such as mutation selection, are often important for growth. For example, central to the development of many tumors is destabilization of the genome and establishment of a mutator phenotype [1], [2]; mutants selected for their ability to proliferate, while surviving environmental stresses, expand their numbers and contribute to tumor growth and progression.

Bacteria often face similar constraints for growth in crowded cell populations. They largely exist within matrix-encased and densely packed communities of cells called biofilms. In biofilms, bacteria develop discrete foci of proliferation, called microcolonies, which become markedly differentiated from the surrounding biofilm. Microcolonies are typically tolerant to most antimicrobial compounds and play an important role in many persistent biofilm infections [3]–[6]. Importantly, cells within microcolonies often proliferate rapidly while other biofilm bacteria are non-dividing and do not increase their biovolume [7]. Studies indicate that early-stage microcolonies of this kind are clonal structures derived from a single cell within the biofilm [8], [9].

Intriguingly, bacteria in biofilms commonly exhibit mutator phenotypes [10], [11] and phenotypic variation [12]–[16], suggesting that mutation and genetic destabilization are an important feature of biofilm development. Factors influencing the evolution of high mutation rates in bacterial populations are a topic of much recent interest [17]–[20], and several recent studies have identified a role for oxidative stress in generating mutation and phenotypic variation among biofilm bacteria [21]–[23]. However the possibility of a role for mutation and genetic change in determining biofilm architecture and microcolony-based growth has not previously been explored and does not feature currently among empirical or mathematical models for biofilm development [7], [24]–[27]. To investigate these potential influences we have examined the role of mutation and mutator phenotypes on microcolony initiation in *Pseudomonas aeruginosa* biofilms. We also designed an experimental procedure using a mutated GFP gene containing a frameshift mutation that results in a loss of GFP expression. This allows real-time observation of fluorescence inducing reversion mutations (FIMS) *in-situ* within biofilms formed by *P. aeruginosa*.

## Results

### GFP-based mutation detection

A GFP-based mutation detection plasmid (pMDGFP) was constructed to allow visualization of *P. aeruginosa* cells that undergo frameshift mutation events in *P. aeruginosa* biofilms. To test the reversion plasmid, *P. aeruginosa* containing plasmid pMDGFP was grown in planktonic batch cultures treated with 0, 8 or 16 µg ml^−1^ of ICR-191, an acridine mutagen that induces predominantly +1 and −1 frameshifts in runs of three or more monotonic GC base pairs [28]. Following overnight incubation with the mutagen, planktonic cells were plated on agar and fluorescent colonies enumerated. The number of fluorescent colonies observed at each dose level is shown in Table 1. We did not detect any FIMS in untreated planktonic cultures that were not exposed to the chemical mutagen ICR-191. We examined approximately 1.45×10^6^ CFU formed by untreated bacteria plated onto agar and did not observe any fluorescent colonies. However, the mutant fractions for *P. aeruginosa* cultures treated with 8 and 16 µg ml^−1^ ICR-191 were 2.0−3.6×10^−5^ and 6.7−10.0×10^−5^ respectively. Our data agree closely with previously published work that has assessed the mutagenicity of ICR-191 using plasmid-based reversion systems in *E. coli* [29], [30].

**Table 1:** Influence of mutagen dose levels on mutation rate in *P. aeruginosa* assessed by pMDGFP FIM fluorescent colony assays.

ICR-191 µg ml^−1^	Cells/plate ( ×10^5^ )	Fluorescent CFU/plate	Mutant fraction
0	1.1	0	<1.45×10^−6^
	5.3	0	
	7.8	0	
8	1.1	4	3.6×10^−5^
	1.9	4	2.1×10^−5^
	4.6	9	2.0×10^−5^
16	1.1	11	1.0×10^−4^
	2.2	16	7.3×10^−5^
	2.7	18	6.7×10^−5^

Data for each of three replicate experiments are shown.

### Mutation detection in *P. aeruginosa* pMDGFP biofilms

**Figure 1:**
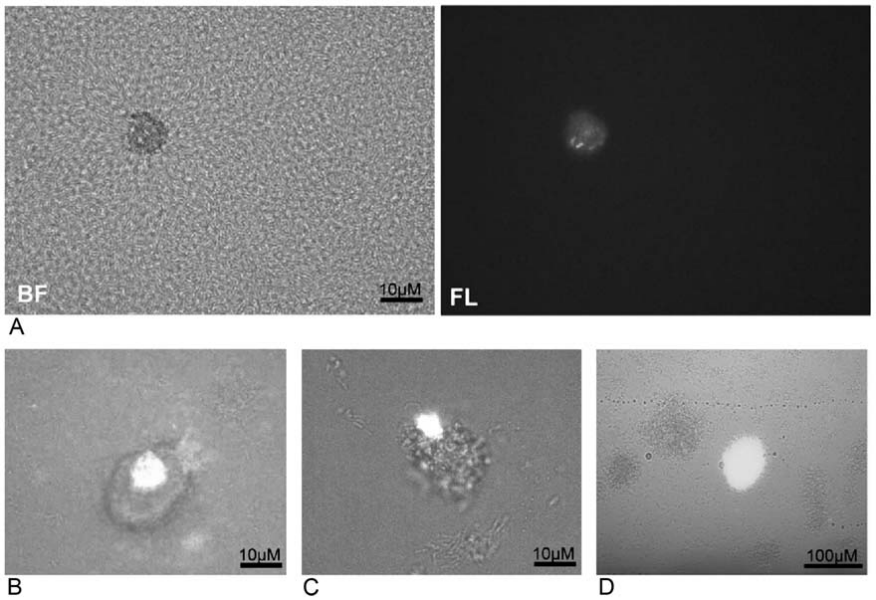
GFP fluorescence-inducing mutations (FIMS) occur during *P. aeruginosa* pMDGFP biofilm development. FIMS were observed exclusively within microcolony structures, but not within unstructured ‘carpet’ regions of the biofilm as in (A), which shows bright field (BF) and fluorescence (FL) images of the same field of view. FIMS were observed as individual GFP-expressing cells (A), or as clusters of GFP cells within microcolonies possibly due to clonal expansion following GPF reversion (B, C). Microcolonies comprised wholly of GFP-expressing cells were occasionally observed (D).

Although we did not observe FIMS in unmutagenized planktonic cultures, we frequently observed FIMS during biofilm culture of *P. aeruginosa* cells containing the pMDGFP plasmid. We observed that fluorescent cells were localized within microcolony structures within the biofilm; in contrast, cells within non-microcolony regions of the biofilm did not contain FIMS in our experiments (Figure 1). Microcolonies contained single fluorescent cells (Figure 1A) or clusters of fluorescent cells (Figure 1B, C). Occasionally, whole microcolonies were observed to be fluorescent, adjacent to microcolonies of similar size and density that were non-fluorescent (Figure 1D), indicating that FIMS had occurred at an early stage during the clonal development of the microcolony. The GFP+1 construct was also delivered to the *P. aeruginosa* chromosome using a mini-Tn7 insertion cassette and delivery vector pMDGFPTn7. Unlike pMDGFP, we were unable to detect brightly fluorescent cells within biofilms when the GFP+1 gene was inserted into the *P. aeruginosa* chromosome in this way. This may be because the single copy chromosomal GFP+1 gene did not result in sufficiently bright fluorescence in the event of a +1 frameshift mutation – thus it is possible that single copy GFP systems are not sensitive enough to detect FIMS in our biofilm system. However, we carried out RNA extraction from 10-day old biofilms and semi-quantitative reverse transcriptase PCR analysis showed that in-frame GFP mRNA was present in the biofilm, but was absent from planktonic cells incubated for the same period in shaking culture (data not shown). We therefore suggest that the GFP+1 chromosomal insertion from pMDGFPTn7 had acquired frameshift mutations within the biofilm, but that insufficient fully folded fluorescent protein was produced to enable fluorescent microscopic observation.

We observed a mean of 1.51±0.25 FIMS mm^−2^ within *P. aeruginosa* pMDGFP biofilms on the glass substratum. Flow cell channels containing WT *P. aeruginosa* biofilms, and channels that were inoculated with suspensions of 1×10^9^ CFU ml^−1^ planktonic pMDGFP *P. aeruginosa*, were also examined and we did not observe FIMS in these control experiments. The mean biovolume of *P. aeruginosa* biofilms at 7 days was 1.88±0.27×10^6^ µm^3^ mm^−2^, containing an estimated 1.4×10^4^ –1.1×10^5^ viable cells [31]. The frequency of reversion mutations in biofilms was therefore 1.3×10^−5^±2.2×10^−6^–1.1×10^−4^±1.8×10^−5^, indicating a minimum increase in mutation frequency of between 6 and 100-fold within the biofilm compared with planktonic cells in our agar plate based assay for mutation detection. However, mutations were observed only within microcolonies, which at 7 days represented 6.2±1.1% of the total biovolume within our biofilms. Therefore microcolony-specific increases in mutation frequency were at least 100-fold in our experiments, and may be up to 1800 fold higher than that observed in planktonic culture. In summary, our data show that bacteria within microcolonies, but not other biofilm bacteria, exhibit elevated frequencies of frameshift mutations within *P. aeruginosa* biofilms.

### Role of mutator phenotypes in microcolony-based biofilm growth

**Figure 2:**
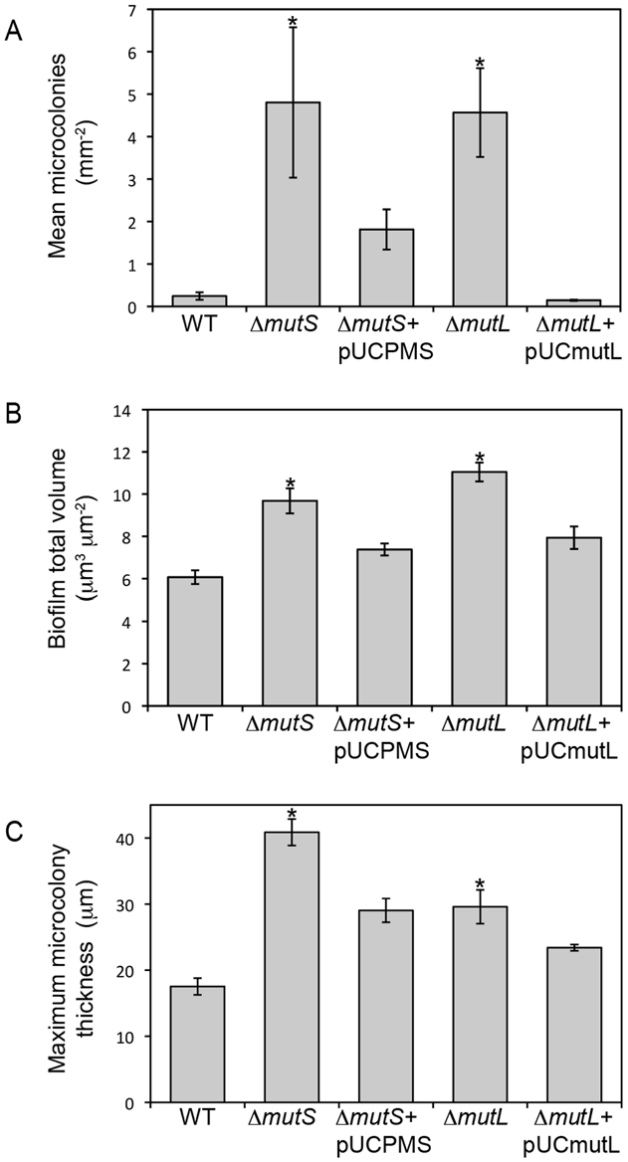
*P. aeruginosa* Δ*mutS* and Δ*mutL* strains exhibit enhanced microcolony initiation and development (A), and also show increased total biofilm volume (B) and maximum microcolony thickness (C). Biofilm development of *P. aeruginosa* PAO1 WT, Δ*mutS*, Δ*mutL*, Δ*mutS*+pUCPMS and Δ*mutL*+pUCmutL grown in continuous culture flow cells over a 10 day period and examined using confocal scanning laser microscopy. Asterisks indicate a significant difference compared with WT or related complemented strain (P<0.05).

**Figure 3:**
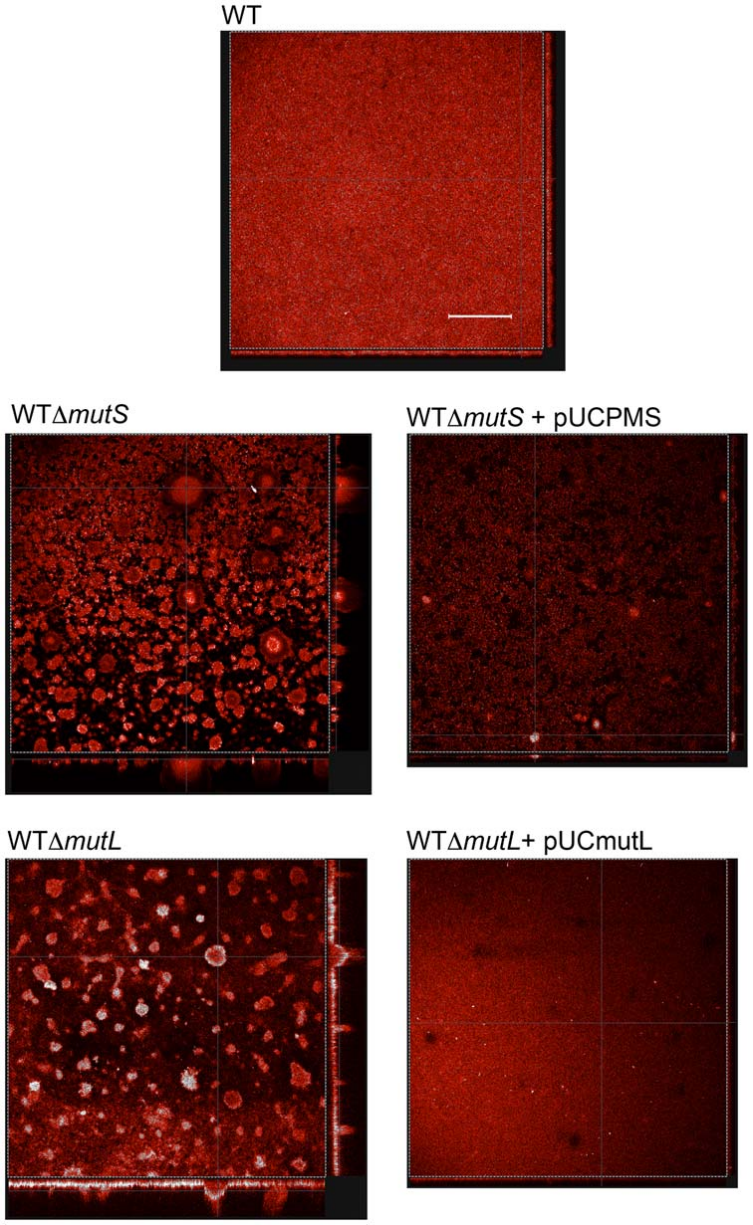
Representative confocal scanning laser microscopy images showing biofilms of *P. aeruginosa* wild-type, Δ*mutS*, Δ*mutL* and complemented strains after 10 days of biofilm development. The central images show a top-down view of the biofilm; side panels are vertical sections. Scale bar represents 150 µm.

Because mutation frequencies were enhanced in *P. aeruginosa* biofilm microcolonies, we examined in more detail whether mutation can influence microcolony growth and development. To determine whether mutation can play a role in microcolony initiation, biofilm growth of *P. aeruginosa* PAO1 WT, Δ*mutS*, Δ*mutL* and complemented mutant strains were compared using confocal scanning laser microscopy (Figures 2 and 3). PAO1 Δ*mutS* cells are unable to repair DNA mismatch errors and exhibit a mutator phenotype with mutation frequencies typically 100 times greater than WT cells, based on a ciprofloxacin resistance assay [32]. We also observed an approximate 100 fold increase in rifampicin resistance mutation frequency in our experiments using the Δ*mutS* strain, as well as for the Δ*mutL* strain constructed in our laboratory, and were able to restore the mutation frequency to wild-type levels by complementation of each of these genes (data not shown). The PAO1 Δ*mutS* and Δ*mutL* strains both formed biofilms with significantly enhanced microcolony growth compared to both the wild-type and respective complemented strains. Biofilms created by the hypermutator strains were significantly larger in total biovolume and maximum microcolony thickness (Figure 2, P<0.05). Thus, mutations in genes that lead to mutator phenotypes in *P. aeruginosa* can enhance microcolony initiation and growth during biofilm culture.

## Discussion

Numerous biomedical and environmental biotechnological applications are affected by the ability of bacteria to grow and adapt to new environments within biofilms. Despite many recent advances in understanding genetic determinants involved in biofilm formation, the growth and development of discrete 3-dimensional microcolony structures within bacterial biofilm communities remains poorly understood. Here we show that microevolutionary processes are involved in the structural development of bacterial biofilms. The work presented here is the first to link mutation with the microcolony architecture of biofilms, and shows that i) bacteria within microcolonies exhibit enhanced mutation frequencies compared with other biofilm bacteria, and ii) bacteria with an elevated mutation frequency can exhibit enhanced microcolony development.

In our experiments, we used a GFP reversion system to detect -1 frameshift mutations *in-situ* within biofilms. Mutations that led to GFP fluorescence occurred specifically within microcolony structures. This observation may suggest that microcolonies, as distinct from non-microcolony biofilm bacteria, may play an important role as foci for genetic adaptation and evolution. For example, strains of bacteria with high mutation frequencies can acquire resistance to antibiotics more rapidly than wild-type strains [33], [34], and also can exhibit enhanced horizontal acquisition of exogenous DNA [35]. Microcolonies may therefore represent key sites of rapid genetic adaptation to antibiotic therapy or other environmental stresses compared with other biofilm bacteria. Documented processes of bacterial dispersal from the interior portions of microcolonies would also provide a mechanism by which new genetic variant cells could be released from the biofilm and colonize new environments [36]–[39]. Moreover, long-term infection of the airways of cystic fibrosis patients by *Pseudomonas aeruginosa* is associated with a series of mutations and specific genetic adaptations to the airway environment [40]. Our data points to microcolony structures as specific sites within biofilms for enhanced genetic adaptation and evolutionary change during chronic respiratory infections such as those associated with cystic fibrosis.

Several possibilities may explain why mutations within biofilms were localized within microcolonies. Microcolonies are foci of cell division and growth within biofilms [7]. Because DNA replication occurs predominantly in dividing cells, microcolonies undergoing DNA replication may accumulate mistakes in DNA replication more rapidly than non-proliferating bacteria. This could lead to the increase in FIMS observed within microcolony structures (Figure 1). However, we did not observe FIMs in rapidly growing planktonic cultures, therefore enhanced DNA replication and cell division in microcolonies cannot alone account for our observations of microcolony-specific mutational events. Mutation could also be a consequence of locally induced DNA-damaging stresses within microcolonies. There are many sources of endogenous DNA damage that can cause mutation within cells, including toxic oxidative products of normal metabolism [41], [42]. Microcolonies generate steep gradients in oxygen and nutrients, which may rapidly generate stresses caused by the accumulation of metabolites. Indeed, several studies have detected endogenous production of reactive oxygen and nitrogen intermediates localized within biofilm microcolonies [43], [44], and oxidative stress has previously been linked to the occurrence of hypermutable *P. aeruginosa* strains in cystic fibrosis infection [45]. Thus it is possible that the unique environment induced by microcolony growth per se may alter mutation and evolution processes occurring within the bacterial population.

**Figure 4:**
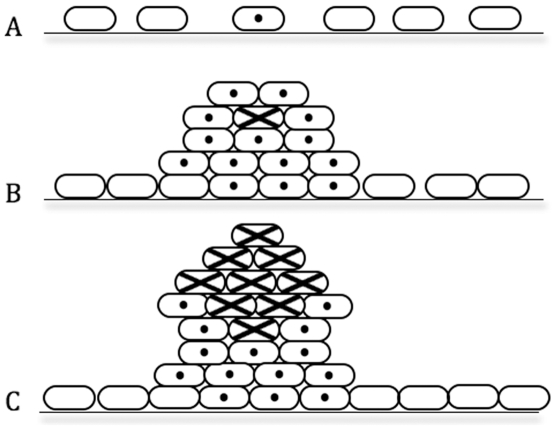
Microevolutionary model for microbial biofilm growth. (A) A bacterial cell attached to a substratum acquires a primary mutation (•) that provides a growth advantage on the surface; (B) mutation selection and clonal expansion occurs; some cells acquire beneficial secondary mutations (×), for example due to exogenous DNA damaging stresses localized to microcolonies; (C) subsequent waves of clonal expansion contribute to microcolony growth and proliferation.

An important question is whether mutation in biofilms may also be a direct cause of microcolony growth. Our data suggest that mutator phenotypes in biofilms can promote microcolony initiation. Experiments involving *P. aeruginosa* Δ*mutS* and Δ*mutL* strains show that enhanced mutation frequencies in *P. aeruginosa* can promote microcolony growth within biofilms (Figures 2 and 3). A possible explanation for this phenomenon is the process of mutation selection, analogous to commonly held models for tumor development (Figure 4). Thus mutated bacterial cells selected for their ability to proliferate on surfaces can expand in number and develop microcolony structures within biofilms. Eukaryotic tumors contain many clonal mutations, i.e. mutations that are present in a proportion of tumor cells and on which selection occurs because they confer a growth advantage. Neoplasms therefore, are composed of ecosystems of evolving clones [46], each competing with other cells in their microenvironment. Within microcolony structures, most of the dynamics of clones and their evolution have not been studied, including mutation rates, fitness effects of mutations within colonies, and competition between co-evolving clones. From the perspective of tumor biology, the capacity to manipulate bacteria to study such processes – particularly the ability to rapidly modify and assess genetic mechanisms – may exceed the analogous capacity in mammalian tumor cells. Thus extension of this work may point the way to a more comprehensive understanding of evolutionary aspects of bacterial biofilm development, as well as providing new perspectives with which to study tumor progression.

## Materials and Methods

### Bacterial strains, plasmids and culture media

*P. aeruginosa* strain PAO1, and two PAO1 mutant strains, Δ*mutS* and Δ*mutL*, were used in this study [32], [47], [48]. Complementing plasmids, pUCPMS [32] and pUCmutL (this study), containing *mutS* and *mutL* respectively were used to restore a wildtype phenotype. The *mutL* region of PAO1 was amplified using MutLF, 5′-CGCGGTAGATCAGCGCCGAGTCGAC-3′; and MutLR, 5′-CCAGGGCAAGCTCTCCATGGGGCC -3′ with PhusionFlash Mastermix (FinnzymesOy, Finland). The purified 2.3Kbp blunt ended fragment was cloned into the SmaI site of pUC24 [49] to create pUCmutL. Transformants were selected from LB agar plates containing 30 µg ml^−1^ gentamicin. The insert orientation was confirmed by PCR using M13 forward primer combined with MutLR primer, and complementation of PAO1Δ*mutL* phenotype shown using the rifampicin resistance method. Batch cultures of *P. aeruginosa* were grown at 37°C with shaking (230 rpm) in Luria-Bertani (LB) broth (Foremedium, UK). Biofilms were cultured using a modified Luria-Bertani broth containing tryptone 1.0 g L^−1^, yeast extract 0.5 g L^−1^, NaCl 10.0 g L^−1^. Spontaneous rifampicin resistance assays were performed using Mueller-Hinton broth and agar (Sigma Aldrich, UK).

Plasmids pMF230 and AKN66 were kindly provided by Michael Franklin, Centre for Biofilm Engineering, Bozeman, MT [50] and Soren Molin, Denmark Technical University [51] respectively. Plasmid pUCPMS was maintained during batch culture and biofilm experiments by the addition of 50 µg ml^−1^ gentamicin; pMDGFP was maintained in *E. coli* and *P. aeruginosa* cultures by the addition of 50 µg ml^−1^ ampicillin and 400 µg ml^−1^ carbenicillin, respectively.

### Mutation detection assay – vector construction and validation

The GFP reversion method has previously been developed for assays of frameshift mutation in eukaryotic and bacterial cells [29], [52]. A +1 frameshift mutation was created within the GFP encoding region of pMF230 and AKN66 using the Phusion™ site-directed mutagenesis kit (FinnzymesOy). Plasmid pMF230 is a high copy number plasmid whereas AKN66 is a mini-Tn7 delivery transposon vector that inserts a single copy of a desired genetic sequence into *P. aeruginosa* [51]. Briefly, mutagenic primers originating within the *GFPmut2* sequence amplified pMF230 and AKN66 and introduced one additional cytosine residue following a native CCC sequence (*GFPmut2* Genbank accession number AF302837, bp 337-339). The primers used were GFP+1F, 5′-CGT GCT GAA GTC AAG TTT GAA GGT GA-3′, and GFP+1R, 5′-TGT CTT GTA GTT CCC CGT CAT CTT T-3′, with the inserted cytosine residue underlined. This produced double stranded linear products that were recircularized by ligation to form plasmids pMDGFP and pMDGFPTn7 and transformed into 5-alpha competent *E. coli* (New England Biolabs, UK). Transformants were selected on LB medium containing 50 µg ml^−1^ ampicillin and a single non-fluorescent colony was selected for DNA sequencing of the *GFPmut2* gene to confirm the presence of the +1 frameshift within pMDGFP. Cells from an overnight culture of *P. aeruginosa* PAO1 were recovered by centrifugation and mixed with 50 ng of purified pMDGFP (Qiagen Plasmid Prep Kit). The cell and DNA mix were suspended in a 300 mM sucrose solution, transformed by electroporation [53], and plated onto LB agar containing 400 µg ml^−1^ carbenicillin. Use of a GFP plasmid-based mutation detection system may enhance the sensitivity of mutation detection due to i) multiple copies of the frameshift-containing GFP gene; pMF230-based plasmids have a copy number of approximately 50–100 per *P. aeruginosa* cell (M. Franklin, unpublished), and ii) presence of a CCCC sequence within the mutated GFP gene; polycytosine repeats can increase sensitivity to frameshift mutations [28].

The chemical mutagen ICR-191 (CAS No. 17070-45-0), Sigma (Poole, UK), was dissolved in DMSO at a concentration of 5 mg ml^−1^. Ten µl of an overnight bacterial culture of *P. aeruginosa*-pMDGFP were inoculated into 10 ml of LB broth in a 50 ml polypropylene tube containing the appropriate amount of mutagen. The culture was grown at 37°C for 18 h in a light-protected shaking incubator (230 rpm). Bacteria were then pelleted by centrifugation, resuspended in 10 ml fresh LB, and incubated for an additional 6 h. A serial dilution of the cells was plated onto LB medium and incubated at 37°C for approximately 16 h. Viable cells were enumerated and plates that were seeded with approximately 1×10^5^ CFU were examined for mutational events leading to GFP fluorescence using epifluorescence microscopy and a 4× objective lens.

Fluorescent colonies induced by ICR-191could be re-streaked to obtain individual isolated fluorescent colonies containing stable revertant GFP-expressing plasmids. By isolating and sequencing plasmid DNA from green fluorescent cells, previous studies using GFP-based reversion detection systems have shown that reversion and GFP production by a relatively small fraction of the total plasmids is sufficient for fluorescence [29].

### Biofilm experiments

*P. aeruginosa* pMDGFP biofilms were grown in continuous-culture flow cells [54] (channel dimensions, 1×4×40 mm; flow rate, 150 µl min^-1^) at room temperature. Each channel was inoculated with 1.0 ml of overnight *P. aeruginosa* culture and incubated for 1 h at room temperature without flow to allow for bacterial attachment to the glass substratum. In order to ensure that an equal density of attached bacterial cells were used to initiate biofilm formation we compared WT and Δ*mutS* strains in our glass flow cell system. After a 2 hr adhesion period, cells were enumerated and no significant difference in attachment was found (data not shown). The flow of culture medium was then started and biofilm growth and development was monitored daily under bright field microscopy. Biofilms were observed at various timepoints when prominent, established microcolonies were visible. Epifluorescence microscopic examination of GFP fluorescence was carried out using an Olympus BX61 microscope equipped with a GFP filter set (BP460-480 nm/BA495-540 nm, Olympus) and captured using a digital camera (Infinity2-2C, http://www.lumenera.com). The intensity of GFP fluorescence observed within *P. aeruginosa* cells was highly variable with camera exposure times ranging from 5 ms to 45 ms.

### Biofilm Quantitation

Confocal laser scanning images of 7 and 10-day old continuous culture biofilms were obtained using a Leica (TCS SP2 MP FCS) upright microscope and 20×/0.70 objective. Images were 1024×1024-pixel resolution and used identical gain, offset and pinhole settings for each data collection point. To enable visualization of cells within the biofilm the medium supply to the flowcell was stopped and each channel was stained for 30 min with 1.0 ml DNA staining solution (5 µM SYTO-9 in LB-10; Invitrogen, U.K.), followed by a period of 10 min with medium flow to wash out excess stain. A single argon laser line was used with excitation wavelength of 488 nm and using an emission filter with a bandpass of 500–600 nm. Three-dimensional rendering and analysis was performed using COMSTAT software [55] on the Matlab platform. COMSTAT analyzed each stack for the total number and volume of microcolonies (>300 pixels) at the substratum (μm^3^), and total biomass (μm^3^). For each image stack, total microcolony volume was expressed as percentage of total biofilm biovolume. Values are calculated means of data from 20 image stacks (5 image stacks from two different channels in two separate experiments). Bacterial cell numbers within biofilms were calculated based on a cell density of 7.5×10^9^–6.0×10^10^ CFU ml^−1^ which was previously determined using individual microcolonies that were measured (volume) and then removed from *P. aeruginosa* biofilms by micropipette [31]; and P. Stoodley, personal communication).

### Mutation frequency assay by activation of the rifampicin resistance gene

Mutation frequencies of all the strains used were estimated using the spontaneous rifampicin resistance method [10]. Briefly, individual 20 ml overnight Mueller-Hinton (MH) broth cultures of each of the *P. aeruginosa* strains were plated onto MH agar plates with and without rifampicin (300 µg ml^−1^) for enumeration following incubation at 37°C for approximately 36 h. All strains were previously susceptible to such concentrations of rifampicin. All experiments were repeated in triplicate and the relevant frequency means calculated.

### Statistical analyses

Statistical analyses of biofilm data from COMSTAT were performed using the Student's t-test and one-way analyses of variance (ANOVA). A significant difference was considered to be p<0.05.
